# Toxicology

**Published:** 1837-04

**Authors:** 


### TOXICOLOGY.

*On the Poisonous Effects of the Berries of the Yew.* By S. Hurt, Esq. Mansfield.

This, as Mr. Hurt justly observes, is a very important case, inasmuch as there exists such discrepancy among the writers on botany and materia medica respecting the poisonous qualities of yew-berries. In the present case there can be scarcely a doubt that death was produced by them, although several other children that ate of the same escaped, and many instances can be adduced of their harmlessness. Five little children were seen under a yew-tree, about eleven o'clock; they returned home a little after twelve, and, while at dinner, one of them, three and a half years old, became sick and vomited, bringing up with its food some portions of yew-berries; convulsions followed, and it died before Mr. Hurt saw it, about two o'clock. The lips were observed to be purplish, and the pupils much dilated. Two days afterwards the body was examined. Purple spots existed on the breast, abdomen, and anterior parts of the legs and arms. The pupils had become much more contracted. The stomach contained, besides potatoes, much mucus and some masticated berries (pulp and seeds) of the yew. There were several red patches on the stomach, and the mucous membrane covering them was quite soft. Mr. Hurt conjectures that the poison resides in the seeds, and is only effectual when these are masticated or so broken as that their substance may come in immediate contact with the stomach. *Lancet, Dec.* 10, 1836.

